# Hypokalaemic periodic paralysis and myotonia in a patient with homozygous mutation p.R1451L in Na_V_1.4

**DOI:** 10.1038/s41598-018-27822-2

**Published:** 2018-06-26

**Authors:** Sushan Luo, Marisol Sampedro Castañeda, Emma Matthews, Richa Sud, Michael G. Hanna, Jian Sun, Jie Song, Jiahong Lu, Kai Qiao, Chongbo Zhao, Roope Männikkö

**Affiliations:** 1 Department of Neurology, Huashan Hospital, Fudan University, Shanghai, 200040 China; 2 Department of Neurology, North Huashan Hospital, Fudan University, Shanghai, 200003 China; 3 MRC Centre for Neuromuscular Diseases, Department of Molecular Neuroscience, UCL Institute of Neurology, London, WC1N 3BG UK; 4 Department of Clinical Electrophysiology, Institute of Neurology, Huashan Hospital, Fudan University, Shanghai, 200040 China; 5 Department of Neurology, Jing’an District Center Hospital of Shanghai, Shanghai, 200040 China

## Abstract

Dominantly inherited channelopathies of the skeletal muscle voltage-gated sodium channel Na_V_1.4 include hypokalaemic and hyperkalaemic periodic paralysis (hypoPP and hyperPP) and myotonia. HyperPP and myotonia are caused by Na_V_1.4 channel overactivity and overlap clinically. Instead, hypoPP is caused by gating pore currents through the voltage sensing domains (VSDs) of Na_V_1.4 and seldom co-exists clinically with myotonia. Recessive loss-of-function Na_V_1.4 mutations have been described in congenital myopathy and myasthenic syndromes. We report two families with the Na_V_1.4 mutation p.R1451L, located in VSD-IV. Heterozygous carriers in both families manifest with myotonia and/or hyperPP. In contrast, a homozygous case presents with both hypoPP and myotonia, but unlike carriers of recessive Na_V_1.4 mutations does not manifest symptoms of myopathy or myasthenia. Functional analysis revealed reduced current density and enhanced closed state inactivation of the mutant channel, but no evidence for gating pore currents. The rate of recovery from inactivation was hastened, explaining the myotonia in p.R1451L carriers and the absence of myasthenic presentations in the homozygous proband. Our data suggest that recessive loss-of-function Na_V_1.4 variants can present with hypoPP without congenital myopathy or myasthenia and that myotonia can present even in carriers of homozygous Na_V_1.4 loss-of-function mutations.

## Introduction

The autosomal dominant channelopathies of the skeletal muscle voltage-gated sodium channel (Na_V_1.4, encoded by *SCN4A*) include myotonia and periodic paralysis (PP)^1–3^ that is often associated with hyper- or hypokalaemia (hyperPP or hypoPP). A patient can manifest with both myotonia and PP, and is diagnosed with paramyotonia congenita (PMC) or hyperkalaemic periodic paralysis (hyperPP) depending on the predominant presentation. Carriers of the same mutation can have different main presentations, even within a pedigree^4,5^. The causative mutations enhance the activity of Na_V_1.4 and predispose the muscle to increased action potential firing that manifests clinically as myotonia. The symptoms can be exacerbated or provoked by raised serum K^+^ levels, which depolarise the potassium equilibrium potential and consequently the resting membrane voltage, thereby promoting Na_V_1.4 activity. In some cases, the increased sodium channel activity and repetitive action potential firing can eventually lead to excess depolarisation of the muscle that then inactivates the sodium channels and prevents further action potential firing, resulting in flaccid paralysis.

Hypokalaemic PP (hypoPP) has a distinct pathomechanism to PMC and hyperPP^1–3,6,7^. The mutations cause a leak current through voltage sensing domains (VSDs) of Na_V_1.4 or skeletal muscle voltage-gated calcium channel Ca_V_1.1^1–3,6–15^. This current is known as the gating pore current and can depolarize the muscle to a level that inactivates the Na_V_1.4 channels and paralyses the muscle, particularly in presence of hypokalaemia that attenuates hyperpolarising currents in the muscle^6,16^. The hypoPP mutations linked to Na_V_1.4 target arginine residues in the fourth transmembrane helix (S4) of three (I-III) of the four homologous VSDs of the channel^6–15^. Mutations affecting S4 arginines in VSD-IV have been shown not to conduct gating pore currents^8,17,18^. Instead, Na_V_1.4 VSD-IV has been implicated as the key VSD regulating fast inactivation^19^, which is often defective for the dominant mutations affecting S4 arginines in VSD-IV^20–22^.

The clinical presentations of autosomal recessive channelopathies of Na_V_1.4 include congenital myasthenia^18,23,24^ and myopathy^25^, and occasionally extreme weakness or paralysis^18,23^. Mutations in these recessive conditions attenuate Na_V_1.4 function. Congenital myasthenia Na_V_1.4 mutant channels show enhanced fast inactivation: the voltage dependence is shifted towards hyperpolarised voltages and the recovery from inactivation is slower than for wild-type channels^18,23,24^. Consequently, upon repetitive stimulation the mutant channels accumulate in the inactive state more than the wild-type channels, which accounts for increased fatigability. In cases with congenital myopathy the functional defects are more variable but one of the *SCN4A* alleles is null^25^, and the weakness in the patients is fixed rather than fluctuating. Curiously, no neurological presentations were reported for heterozygous carriers of null variants^25^.

Myotonia is characteristically considered a distinguishing clinical feature in determining a diagnosis of either hyperPP or hypoPP^26^, consistent with distinct pathomechanisms and opposite dependence on extracellular potassium concentration. There are rare reports, however, of patients presenting with both hypoPP and myotonia^21,27^. We describe here a consanguineous Chinese patient presenting with episodes of paralysis, associated with hypo- or normokalemia, and with myotonia. Next generation sequencing of the proband identified a homozygous mutation p.R1451L affecting the second S4 arginine in VSD-IV. A second unrelated heterozygous p.R1451L pedigree presented with hyperPP and myotonia. Our functional analysis of the mutant channel describes the pathomechanisms underlying the recessive hypoPP and the rarely reported co-presentation of hypoPP and myotonia.

## Results

### Clinical features

#### Family 1

**Figure 1:**
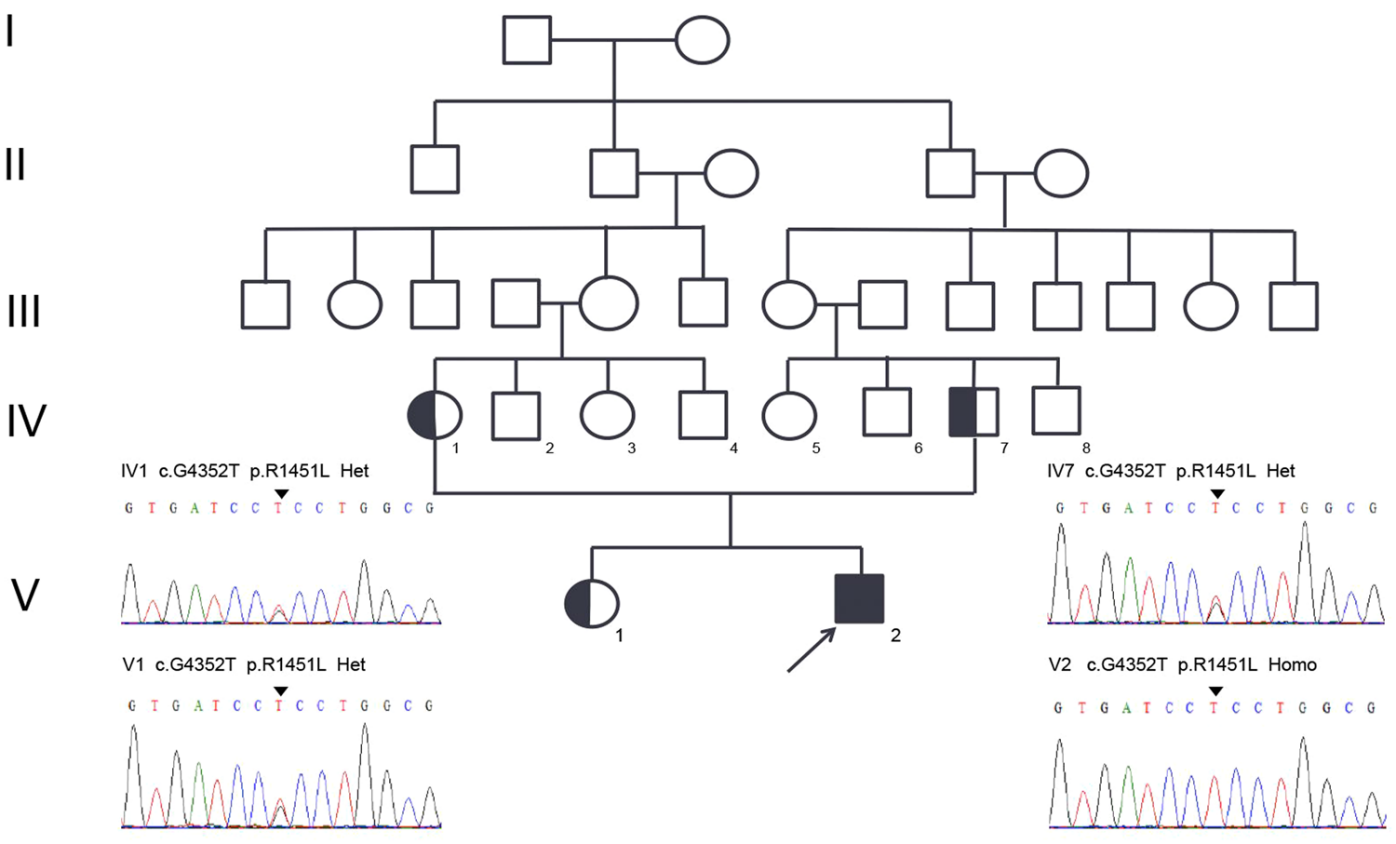
The Pedigree of Family 1. Co-segregation analysis revealed that the proband carried a homozygous p.R1451L mutation in Na_V_1.4, while the consanguineous parents and the sister carried the variant in heterozygosis.

The proband is a 19-year-old Chinese male presenting with a 15 year history of recurrent quadriplegia associated with hypokalaemia or normokalaemia (Fig. 1V2). He experienced his first episode after a high fever at the age of 4. After he turned 7 the episodes occurred 2–3 times per year and usually lasted for several hours to 2 days with spontaneous full recovery. The attacks became more frequent when he was 16. Generalised limb muscle weakness occurred once or twice per month and isolated lower limb weakness 4–5 times per month. The ability to swallow and speak was well preserved. Triggers include rest after a period of vigorous exercise, exposure to cold, upper respiratory infection, and maintenance of a fixed posture. Serum potassium level was 2.8, 3.3 and 4.0 mmol/L (normal range: 3.5–5.5 mmol/L) measured in three distinct episodes of paralysis. None of oral administration with potassium, carbamazepine and topiramate can reduce the attacks or ameliorate the severity. Additionally, physical examination revealed a generalised increase in muscle bulk, especially in bilateral quadriceps and calf muscles. The patient didn’t complain of clinical myotonia, but a decreased ability to relax after a forced eyelid closure was demonstrated. Neither grip myotonia nor percussion myotonia was detected. Muscle strength and the tendon reflex were normal. After coenzyme Q10 administration for 2 months (200 mg/day), the patient reported that recurrent muscle weakness was greatly relieved. The effect of coenzyme Q10 treatment on eyelid myotonia was not assessed.

Next generation sequencing revealed the homozygous p.R1451L mutation in the skeletal muscle Na_V_1.4 sodium channel. No other causal mutations were detected in a panel of 10 ion channel genes and 245 primary myopathies/muscular dystrophies-related genes (Supplementary Table S1).

The parents are consanguineously married carriers (Fig. 1 IV1 and IV7) of the heterozygous p.R1451L mutation. Their running ability is unaffected and they do not display symptoms of clinical myotonia although the muscle volume is relatively increased. A 22 years old sister with a heterozygous p.R1451L mutation complained of muscle stiffness after a short rest. The father had no complaints of muscle weakness. Clinical PP was not observed in the heterozygous carriers of p.R1451L.

#### Family 2

A 23 year old man presented to the national referral centre for skeletal muscle channelopathies in the UK with symptoms of episodic muscle weakness. His birth history, motor milestones and childhood were normal with no specific symptoms. At the age of 18 years after a period of prolonged sitting he experienced bilateral leg weakness that lasted for several hours and spontaneously resolved. From this point he experienced recurrent episodes of limb muscle weakness and at the time of referral these were occurring daily. They could occur at any time of the day although a significant number occurred first thing in the morning with less frequent episodes during the night. Symptoms lasted typically from 10 mins to a few hours and could be exacerbated by cold weather, exercise or prolonged rest. He did not identify any specific food triggers. Three episodes all occurring from sleep had been severe enough to warrant hospital attendance. Serum potassium was recorded within the normal range on each occasion. In addition to the attacks of weakness he reported his hands could become “stuck”, particularly in cold weather.

Examination revealed him to have a muscular physique, which was notable relative to a general lack of exercise. There was mild hand and grip myotonia that was exacerbated by repetition, but the remainder of the exam was unremarkable. He reported a family history of affected father with similar symptoms although had no regular contact with him. Although the diurnal pattern is reminiscent of hypoPP, all other clinical features were considered consistent with hyperPP and the patient was treated with a combination of acetazolamide 500 mg BD and bendroflumethiazide 5 mg OD. This reduced although did not abolish his attacks of paralysis. There was no effect on myotonia.

Genetic analysis identified the heterozygous *SCN4A* mutation p.R1451L in both the proband and his father.

### Electrodiagnostic studies

Electromyographic (EMG) studies of the proband in family 1 demonstrated myotonic discharges in a number of tested muscles (Table 1; Deltoids, Flex Carpi Rad, Vastus Med, Gastroc caput med and Tibialis anterior). Of note, poor recruitment of muscle unit potential was demonstrated in the rectus abdominis and Vastus Med. Myotonic discharges are documented in some selected muscles for the parents and the sister (Table 1). Repeat EMG was not performed following administration of Coenzyme Q10.

**Table 1:** Myotonic discharges identified in EMG study.

	Pedigree	Father	Mother	Sister
Masseter	—	—	—	—
Sternocleidomast	—	myotonia	—	—
Deltoids	myotonia	—	—	—
Biceps	—	myotonia	myotonia	myotonia
Flex Carpi Rad	myotonia	myotonia	myotonia	—
Inteross dors I	—	myotonia	—	myotonia
Rectus abdominis	—	—	—	—
Iliopsoas	—	—	—	—
Vastus med	myotonia	myotonia	—	—
Gastroc caput med	myotonia	myotonia	—	—
Tibialis anterior	myotonia	myotonia	myotonia	—

**Figure 2:**
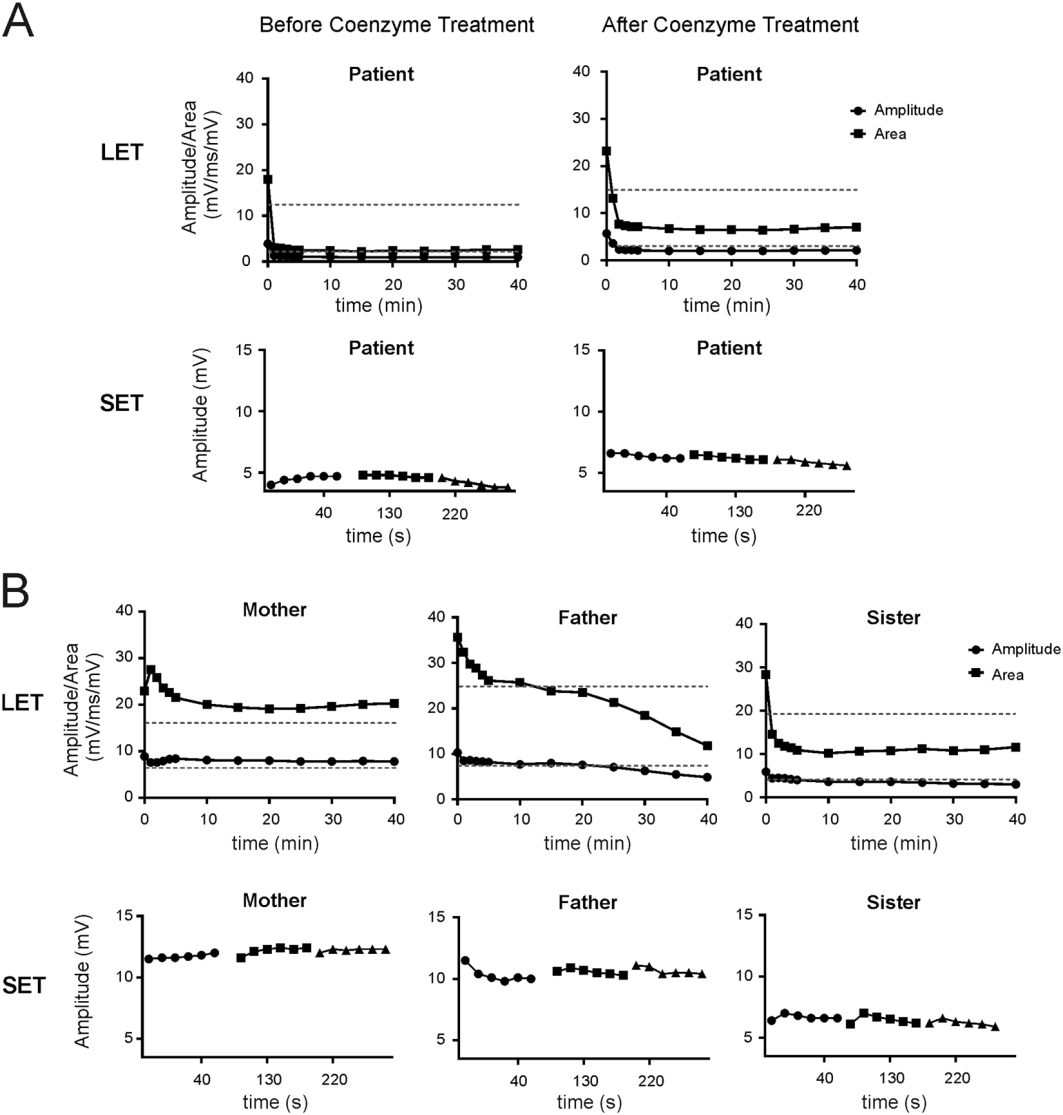
Electrodiagnostic studies of homozygous and heterozygous p.R1451L carriers. (**A**) A significant CMAP amplitude decline was demonstrated in the homozygous patient (left). An increase of baseline amplitude was demonstrated for the proband after Coenzyme Q10 treatment (right). (**B**) A significant CMAP amplitude decline was demonstrated in the father and the sister, but not the mother.

Long exercise test (LET) revealed a significant decline of >30% of baseline amplitude value for the proband (Fig. 2A). After two months oral administration of Coenzyme Q10, compound muscle action potential (CMAP) decrement >30% occurred at the same time, but the baseline value of long exercise test increased from 3.9 mV to 5.7 mV in abductor digiti minimi (ADM) muscle (Fig. 2A).

For the father and the sister, a significant decline of CMAP amplitude in LET was observed 25 minutes and 10 minutes after exercise, respectively. For the mother, the CMAP amplitude and area slightly declined and did not reach a 30% threshold (Fig. 2B).

The proband in family 2 had evidence of myotonic runs in the first dorsal interosseous and tibialis anterior. A long exercise test was positive for periodic paralysis with a decrement of CMAP amplitude from the baseline of 67%.

### Analysis of gating pore currents

**Figure 3:**
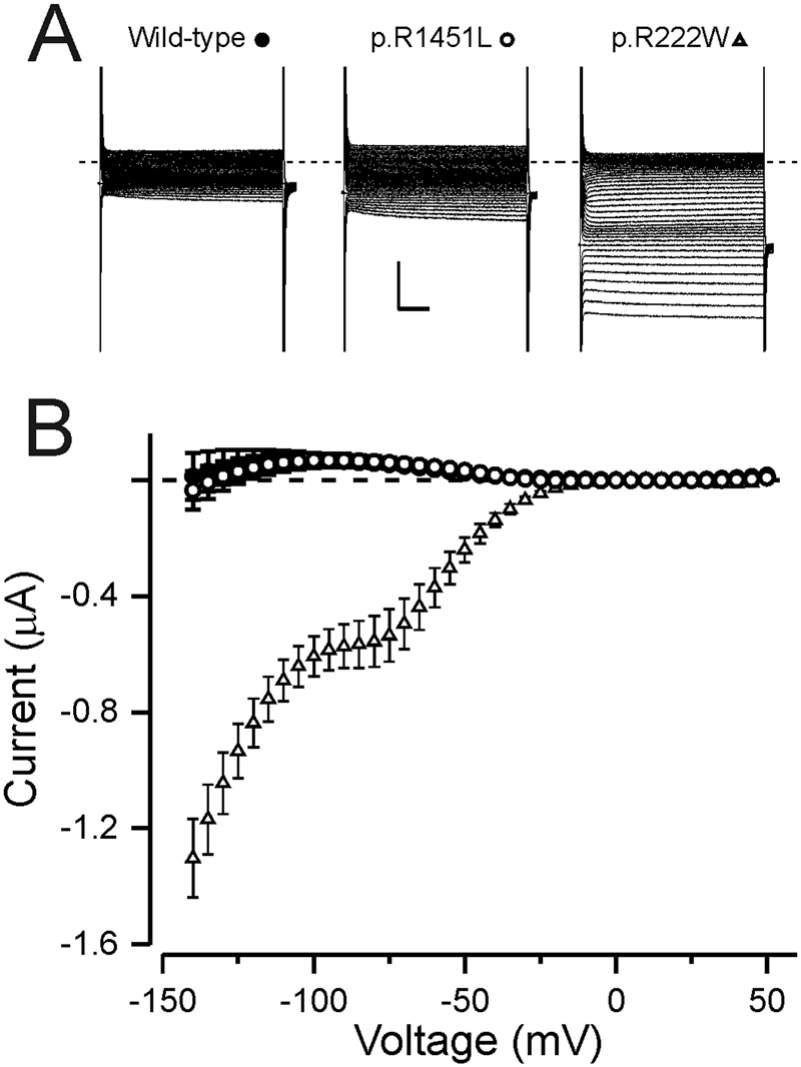
Na_V_1.4 R1451L does not conduct gating pore currents. (**A**) Representative current traces of wild-type and p.R1451L channels expressed in *Xenopus* oocytes. The response to test voltages ranging from −140 mV to +50 mV in 5 mV increments is shown. Holding voltage was −100 mV. The capacitive currents are clipped. Scale bars are 50 ms (x) and 0.5 µA (y) (**B**) Leak subtracted current plotted against the test voltage for wild-type (solid circles, n = 5) and p.R1451L (open circles, n = 10) channels in 50% guanidinium solution. The current of p.R1451L did not differ from the wild-type (I_−80_ p.R1451L 64 ± 18 nA *vs*. WT 58 ± 16 nA). As positive control, the current response of hypoPP mutant Na_V_ 1.4 R222W (triangle, n = 3) is shown (I_−80_ = −567 ± 81 nA).

As hypoPP is associated with gating pore currents caused by mutations that affect S4 arginines we first analysed if p.R1451L mutant channels conducted gating pore currents using the *Xenopus laevis* oocyte expression system. To isolate the gating pore currents the main pore was blocked with 1–2 µM tetrodotoxin. The amplitude of the gating pore current of S4 arginine mutant channels is increased when guanidinium acts as charge carrier^13,28^. However, when 50% of the extracellular sodium was substituted by guanidinium, steady state current amplitude did not differ between p.R1451L and wild-type channels at physiological voltages (Fig. 3), whereas currents in known hypoPP mutant p.R222W^29^ were significantly larger (one way ANOVA, Dunnet multiple comparisons, compared at −80 mV). These data suggest that p.R1451L channels do not conduct gating pore currents.

### Analysis of main pore currents

**Figure 4:**
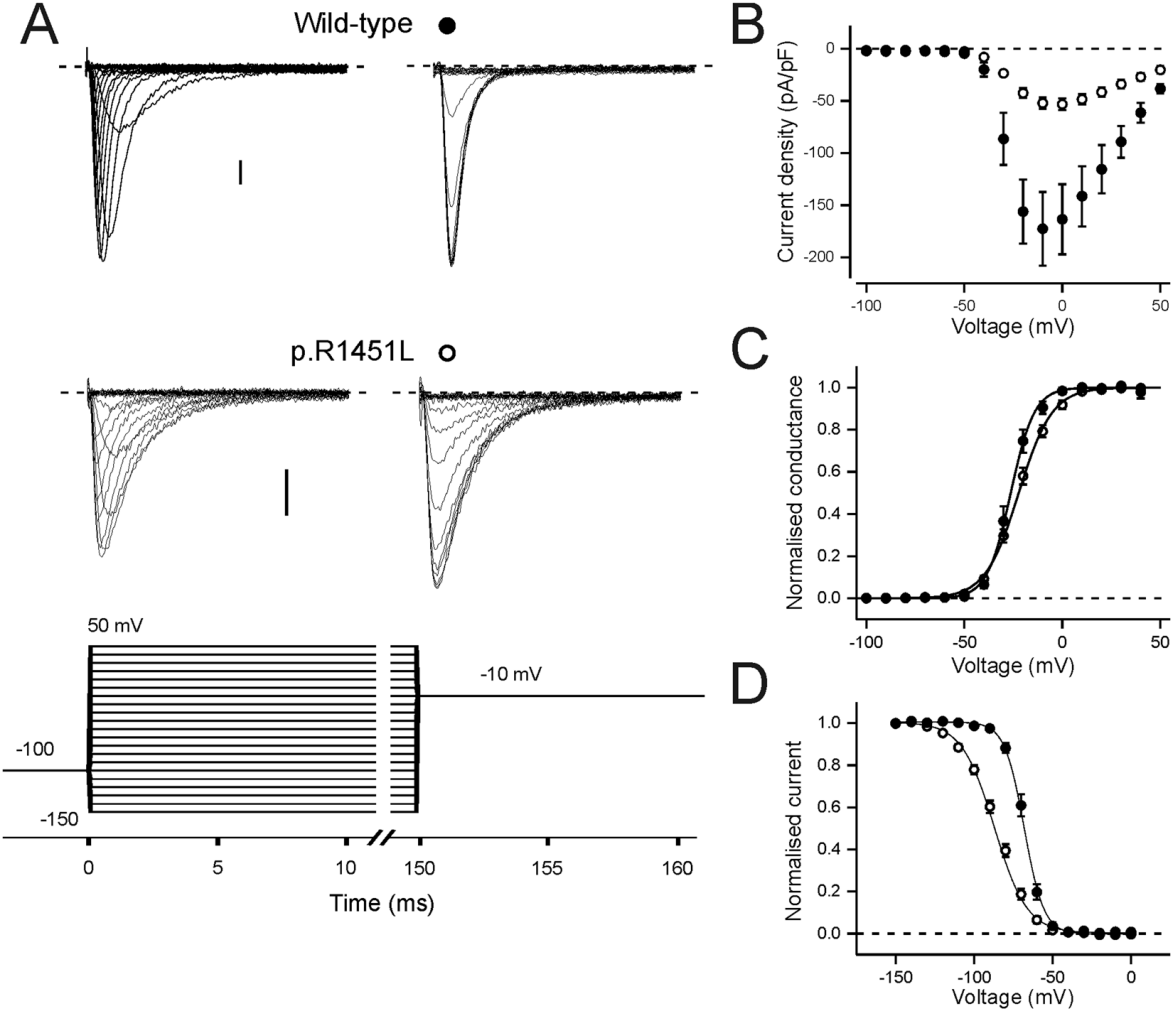
Voltage dependence of activation and fast inactivation. Number of experiments is given in Table 2. (**A**) Representative current traces of wild-type and p.R1451L channels in response test voltages ranging from −100 mV to +50 mV in 10 mV increments, followed by a tail pulse to −10 mV. Holding voltage was −100 mV. Y-axis scale bar is 20 pA/pF. The voltage protocol is shown below. (**B**–**D**) Wild-type data is shown in solid symbols, p.R1451L data in open symbols. (**B**) Peak current density is plotted against the corresponding test voltage. (**C**) The current data from A is converted to conductance. (**D**) Peak tail current at −10 mV is plotted against the test voltage. For (**C**) and (**D**), data from individual cells was normalised to top and bottom amplitude of a Boltzmann fit. Mean ± SEM of normalised data is shown. Solid lines represent the fit of a Boltzmann equation to the mean data.

**Figure 5:**
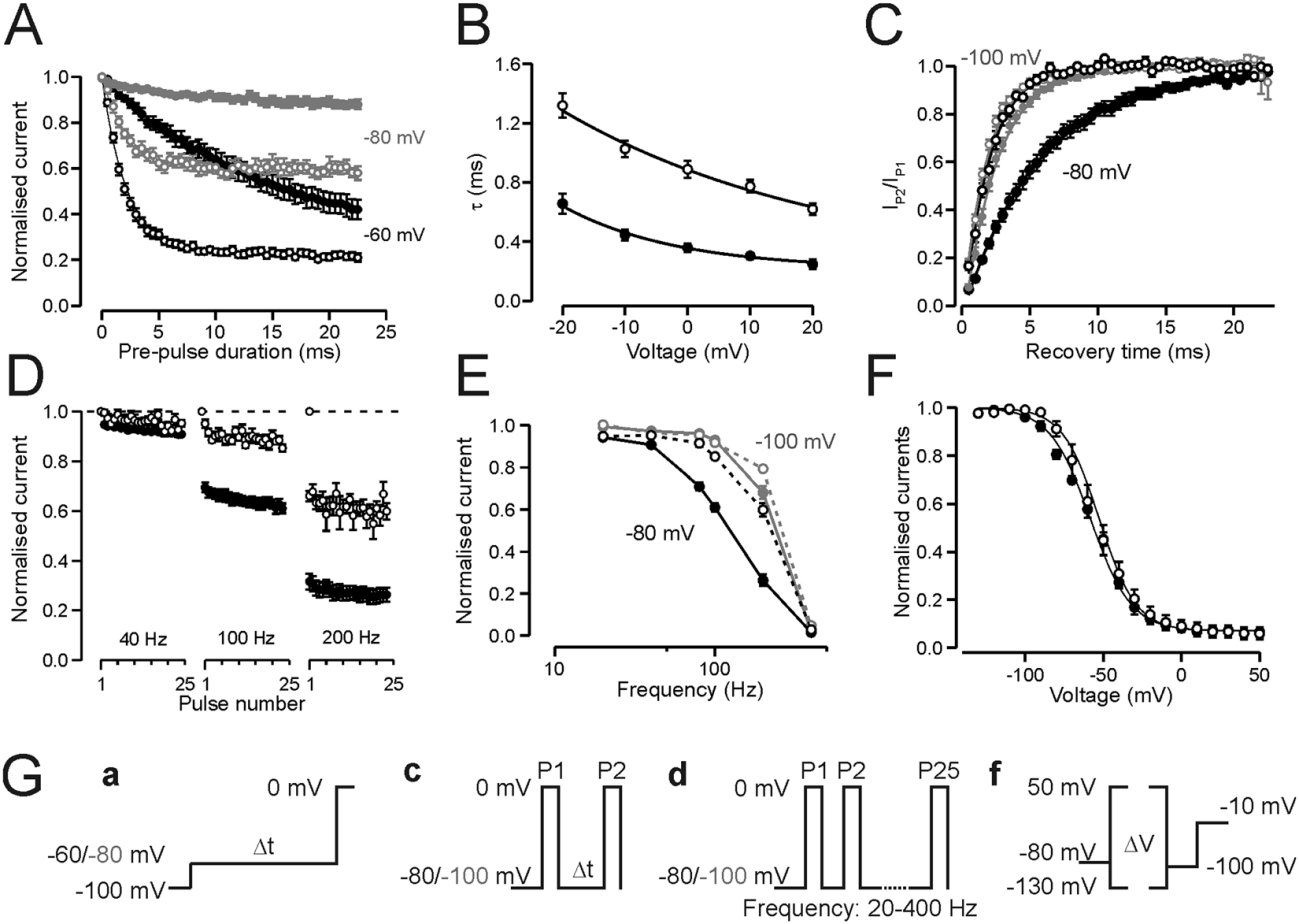
Inactivation properties. Wild-type data is shown in solid symbols, p.R1451L data in open symbols. Number of experiments is given in Table 2 and voltage protocols are described in the methods and shown in (**G**). (**A**) Rate of closed state inactivation at −80 mV (grey) and −60 mV (black) from V_h_ −100 mV for wild-type and p.R1451L channels. Current in response to test voltage is plotted against the time at pre-pulse voltage. Data are normalised to maximum current in response to test pulse. (**B**) Time constant of open state inactivation at voltage range −20 mV to +20 mV. (**C**) Rate of recovery from inactivation at −80 mV (black) and −100 mV (grey) for wild-type and p.R1451L channels. Current in response to second test pulse is divided by current in response to first pulse and plotted against the time at recovery voltage. (**A**–**C**) Solid lines represent the fit of an exponential function to the mean data. (**D**) Peak current amplitude in response to 2 ms pulse to 0 mV is plotted against the pulse number when the train of pulses was applied at 40 Hz, 100 Hz and 200 Hz frequency for wild-type (5–8 cells/frequency) and p.R1451L (5–14 cells/frequency) channels. Current is normalised to the amplitude of the first test pulse. V_h_ = −80 mV (**E**) Normalised current amplitude of the 25^th^ pulse is plotted against the frequency of the pulse train. V_h_ was −80 mV (black) or −100 mV (grey). (**F**) Voltage dependence of slow inactivation (V_h_ = −80 mV). Current in response to test voltage is normalised to peak amplitude of Boltzmann fit and plotted against the pre-pulse voltage. Solid lines represent the fit of a Boltzmann equation to the mean data. (**G**) Voltage protocols for rate of closed state inactivation (a), rate of recovery from inactivation (c), train of pulses (d) and slow inactivation (f). Rate of open state inactivation was measured using the protocol in Fig. 4.

We studied the main pore sodium current of the p.R1451L mutant in HEK293 cells (Figs 4, 5, Table 2). Consistent with previous analysis of p.R1451L properties^21^, the peak current amplitude of the mutant channel was significantly reduced compared to the wild-type channel (Fig. 4A), the voltage of half-maximal fast inactivation was shifted to more hyperpolarized voltages and the slope was more shallow (Fig. 4C). We didn’t observe changes in the voltage of half-maximal channel activation, although the slope of p.R1451L activation was significantly more shallow than for wild-type channels (Fig. 4B). The voltage dependence of activation and fast inactivation were similar when the holding voltage (V_h_) was −80 or −100 mV (Table 2).

**Table 2:** Comparison of the biophysical properties of Na_V_.1.4 WT and p.R1451L channels at two different holding potentials (V_h_).

V_h_ −80 mV	WT	n	R1451L	n	p-value
Current density at 0 mV (pA/pF)	−166.7 ± 28.2	23	−28.5 ± 3.0	20	<0.001
**Activation**					
V_1/2_ (mV)	−19.8 ± 0.8	22	−21.3 ± 1.1	19	0.28
*K* (mV)	5.8 ± 0.3		8.4 ± 0.3		<0.001
**Fast Inactivation**					
V_1/2_ (mV)	−65.6 ± 0.7	22	−87.7 ± 1.4	20	<0.001
*K* (mV)	5.3 ± 0.1		11.6 ± 0.3		<0.001
**Open State Inactivation**					
τ at 0 mV (ms)	0.35 ± 0.03	22	0.90 ± 0.06	15	<0.001
**Recovery from fast inactivation**					
τ at −80 mV (ms)	6.7 ± 0.8	12	2.0 ± 0.1	6	<0.001
**Slow Inactivation**					
V_1/2_ (mV)	−56.9 ± 0.9	10	−53.8 ± 3.0	5	0.33
*K* (mV)	14.7 ± 0.6		11.3 ± 0.6		<0.01
**V**_**h**_ **-100 mV**					
Current density at 0 mV (pA/pF)	−163.5 ± 33.6	6	−56.0 ± 5.8	19	<0.05
**Activation**					
V_1/2_ (mV)	−26.2 ± 1.8	6	−22.6 ± 1.1	18	0.11
*K* (mV)	5.5 ± 0.4		7.8 ± 0.2		<0.001
**Fast Inactivation**					
V_1/2_ (mV)	−67.7 ± 1.1	6	−85.4 ± 1.4	19	<0.001
*K* (mV)	5.5 ± 0.4		10.3 ± 0.2		<0.001
**Open State Inactivation**					
τ at 0 mV (ms)	0.36 ± 0.03	6	0.88 ± 0.06	18	<0.001
**Recovery from fast inactivation**					
τ at −100 mV (ms)	2.4 ± 0.2	7	1.6 ± 0.1	15	<0.001
**Closed State Inactivation**					
τ at −60 mV (ms)	16.8 ± 2.2	6	2.1 ± 0.2	11	<0.001
τ at −80 mV (ms)	8.5 ± 1.6	5	2.2 ± 0.3	7	<0.01

We further analysed the defective fast inactivation at two physiological holding voltages (Fig. 5, Table 2). With V_h_ −100 mV, the onset of closed-state inactivation at −60 mV and −80 mV was significantly accelerated for p.R1451L channels compared to wild-type channels, consistent with the left-shift in the voltage dependence of fast inactivation (Fig. 5A). In contrast, the onset of p.R1451L open-state inactivation was decelerated compared to wild-type channels in the studied voltage range −20 mV to +20 mV (Fig. 5B). The rate of closed-state inactivation at −60 mV was accelerated for wild-type channels when the holding voltage was −80 mV, compared to data with V_h_ = −100 mV (Supplemental Fig. S1), but was not analysed for p.R1451L channel as at this V_h_ more than 50% of the channels are already inactivated. The rate of open-state inactivation was unaffected by the holding voltage (Table 2). The rate of p.R1451L channel recovery from inactivation was 3 times faster than for the wild-type channel at −80 mV (p.R1451 L τ = 1.9 ms *vs* τ = 6.1 for wild-type channel, Fig. 5C). At −100 mV the rate of recovery was accelerated for both channels and although the fold difference was reduced, the recovery of p.R1451L channels was still significantly faster than for wild-type channels (p.R1451L τ = 1.6 ms *vs* τ = 2.4 for WT, Fig. 5C).

A prediction of the accelerated recovery from inactivation and the reduced rate of open-state inactivation of p.R1451L channels is that upon repetitive high-frequency stimulation the availability of the mutant channels reduces less than for wild-type channels. Consistently, when a 2 ms pulse to 0 mV was given from a holding voltage of −80 mV at frequencies 40–200 Hz the current declined more for wild-type than for mutant channels (Fig. 5D,E). When V_h_ was −100 mV the difference in availability between p.R1451L and wild-type channels was only observed at very high frequency (200 Hz), probably reflecting the reduced difference in the rate of recovery from inactivation of both channels at this voltage.

Finally, the p.R1451L mutation did not alter the voltage of half-maximal slow inactivation of the Na_V_1.4 channel although the slope was slightly shallower for p.R1451L channels (Fig. 5F, Table 2).

## Discussion

We present heterozygous and homozygous cases of the same *SCN4A* mutation p.R1451L with differing clinical features. Our homozygous case experienced hypoPP and myotonia. These conditions have distinct molecular pathomechanisms and are rarely reported to co-exist in an individual^26^, so much so that the presence of myotonia in a patient is often used to exclude diagnosis of hypoPP. Heterozygous carriers of this mutation, presented here and in a previous study^21^, fall within a myotonia-hyperPP spectrum of disorders. The myotonia of the heterozygous carriers can be triggered by cold^21^ and exacerbated by repetition, consistent with a diagnosis of PMC. An episode of hypokalaemia-associated PP was reported in a p.R1451L heterozygous carrier^21^.

The p.R1451L channel displays gain-of-function-properties that explain the myotonia and hyperPP in carriers of p.R1451L: the rate of open state inactivation was reduced and the rate of recovery from inactivation was enhanced at physiological voltages. Consequently, p.R1451L channels showed improved channel availability upon high frequency repetitive simulation. Accelerated recovery from inactivation or changes in channel availability upon repetitive stimulation were not previously reported for this mutant^21^, probably reflecting the fact that these were measured only at very hyperpolarized voltages where the recovery rate may saturate for both p.R1451L and wild-type channels. Heterozygous p.R1451L carriers show a range of predominant presentations within the PMC-hyperPP spectrum. Similar range of manifestations has been reported for other Na_V_1.4 mutations^4,5^ but the mechanism determining the predominant manifestation remains to be characterized.

The changes in the properties of the p.R1451L mutant resemble channels with mutations that affect the outermost arginine residue in the S4 helix of VSD-IV, R1448^20,22^. These mutations impart gain-of-function properties to Na_V_1.4 by reducing the rate of open state inactivation and by accelerating the rate of recovery from inactivation, but they also left-shift the voltage dependence of fast inactivation, as described here for p.R1451L. In accordance, the heterozygous carriers of these mutations are within the PMC-hyperPP spectrum, similar to heterozygous carriers of the p.R1451L mutation.

The predominant symptom of the homozygous carrier of p.R1451L carrier is PP associated with hypo- or normokalaemia. Gating pore currents could not be detected in p.R1451L channels, consistent with existing data for p.R1451H channels^17^, suggesting that depolarisation of the muscle by gating pore currents does not underlie hypoPP in our patient.

It has been shown that recessive Na_V_1.4 loss-of-function mutations can present with episodes of paralysis^18^ or with “lifelong episodic generalized weakness”^23^. These mutations are associated with congenital myasthenia and cause enhanced fast inactivation and affect arginine residues R1454^18^ and R1457^23^, both adjacent to R1451 in the S4 helix of VSD-IV. Mutation p.R1451L also enhances channel inactivation, resulting in increased susceptibility of the muscle to depolarisation-induced reduction in Na_V_1.4 channel availability. Thus, a small depolarisation, potentially induced by hypokalaemia, may reduce p.R1451L channel availability to a level insufficient to sustain muscle tone in the homozygous carrier, resulting in flaccid paralysis. Other loss-of-function features, such as reduced current density contribute towards the reduced channel availability and the weakness in the homozygous patient. Also, although we did not detect changes in voltage of half-maximal slow inactivation, a reduction in the rate of recovery from slow inactivation at −120 mV was recently reported for p.R1451L channels^21^. This loss-of-function feature may also contribute towards the clinical presentation.

However, unlike the carriers of other homozygous or compound heterozygous Na_V_1.4 mutations that display PP, the homozygous carrier of p.R1451L does not display myasthenic symptoms. Despite left-shifted voltage dependence of fast inactivation the recovery rate of p.R1451L channels from fast inactivation is enhanced, contrary to that described for congenital myasthenia mutations. Consequently, upon repetitive stimulation, congenital myasthenia variants accumulate more in the inactivated state than wild-type channels^18,23,24^, while p.R1451L channels accumulate less (Fig. 5D,E). Enhanced accumulation in the inactivated state has been linked with enhanced reduction in CMAP upon repetitive stimulation in carriers of the myasthenic variants^23,24^. The rate of open-state inactivation is reduced for all p.R1454W, p.R1457H and p.R1451L mutant channels, suggesting that this feature cannot qualitatively explain the absence of myasthenic syndromes in the homozygous p.R1451L patient. Thus, our data pinpoints the rate of recovery from fast inactivation as a key determinant of clinical presentation in these cases and highlights R1451 as a pivotal residue controlling the speed of recovery from inactivation.

**Figure 6:**
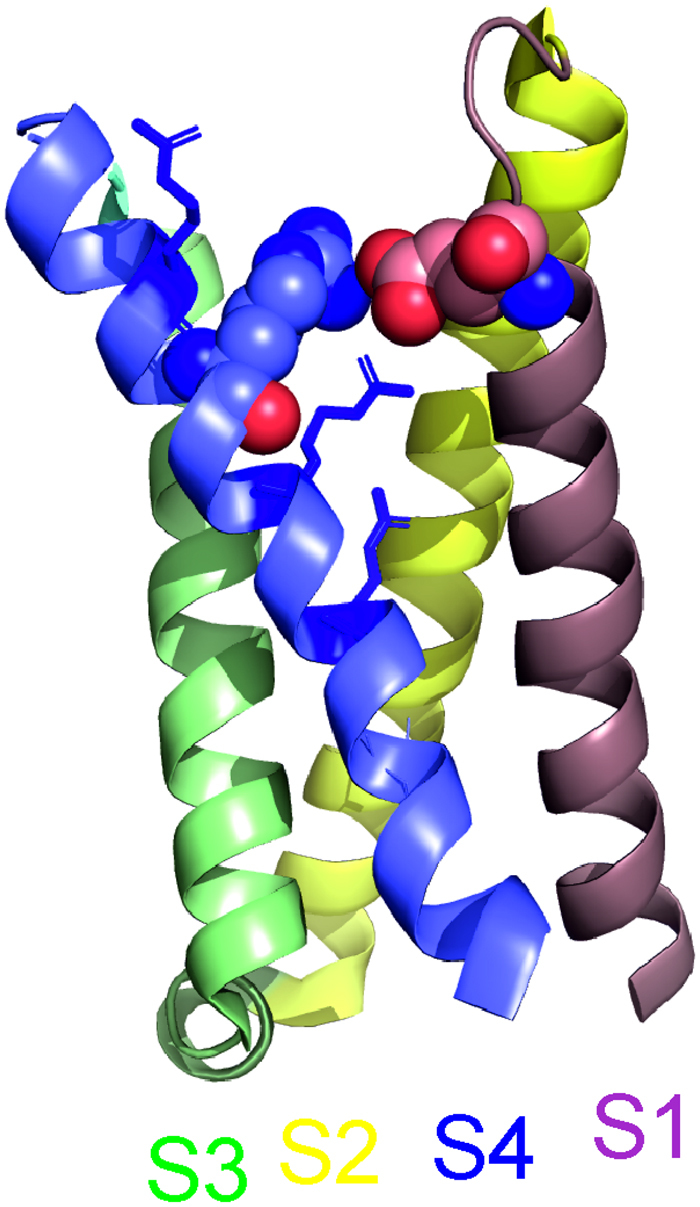
Location of R1451 in the “up” conformation of Na_V_1.4 VSD-IV. In the cryo-EM structure of electric eel Na_V_1.4 (protein data bank ID 5XSY) the residue corresponding to Na_V_1.4-R1451 (blue spheres) is located in proximity to the residue corresponding to Na_V_1.4-E1373 (pink spheres) in the S1-S2 loop of VSD-IV. The distance between the guanidino- and delta-carbons of R1451 and E1373, respectively, is 5.78 Å. Arginines corresponding to R1448, R1454 and R1457 are shown in blue sticks. The structure is illustrated with the PyMOL Molecular Graphics System, Version 2.0 Schrödinger, LLC.

The structure of electric eel Na_V_1.4^30^ suggests that a mutation of R1451 disrupts its electrostatic interaction with E1373 (Fig. 6). This interaction may stabilise the ‘up’-state of VSD-IV captured in the Na_V_1.4 structure, and, in accordance, the p.R1451L mutation may destabilize the ‘up’-state and allow hastened recovery of the voltage sensor to the resting state, accounting for the faster rate of recovery from inactivation. Consistently, neutralising or charge-reversing mutations of E1373 also accelerate the rate of recovery from inactivation^31^.

An episode of hypoPP was reported for a single heterozygous p.R1451L carrier^21^ suggesting that, in rare cases, heterozygous loss-of-function of Na_V_1.4 may increase susceptibility to hypokalaemia induced PP. However, heterozygous carriers of Na_V_1.4 loss-of-function variants, including null variants, have been reported asymptomatic^18,23–25^ suggesting that loss-of-function affecting a single allele does not reduce Na_V_1.4 availability sufficiently to cause fixed or fluctuating weakness. Consistently, mice with heterozygous Na_V_1.4 null allele do not show increased susceptibility to periodic paralysis^32^. This suggests that loss-of-function features in a single allele very rarely underlie neurological presentations, at least in the absence of other factors affecting muscle excitability.

Reports of bi-allelic gain-of-function variants are rare. In a carrier of the homozygous PMC mutation, p.I1393T, no episodes of PP associated with low potassium were reported^33^. Instead, the patient suffered from severe myotonia. Mice that carry homozygous gain-of-function mutations did not survive beyond day two, with noted breathing difficulties likely associated with breathing muscles^34^ or showed greatly impaired embryonic or early postnatal survival^35^, potentially explaining the rarity of such cases in humans. Thus, it is unlikely that the gain-of-function features are solely responsible for the PP associated with hypokalaemia in the homozygous carrier of p.R1451L mutation. However, these may contribute towards the clinical presentation. Cold, that slows the rate of inactivation of both WT and p.R1451L channels^21^, was reported as one of the triggers of weakness in the homozygous carrier, supporting a role for this gain-of-function feature in the clinical presentation.

Our data are consistent with a model where in heterozygosis the gain-of-function features of p.R1451L cause symptoms on the PMC-hyperPP overlap, while in homozygosis the loss-of-function features underlie hypo-/normoPP. However, available clinical detail cannot confirm the pathomechanism of the weakness in the p.R1451L homozygotic patient and it is possible that gain-of-function properties of p.R1451L contribute to this manifestation. Other intrinsic and extrinsic factors may also influence the range of clinical manifestations in the heterozygous and homozygous carriers of this mutation. Co-existence of recessive loss-of-function properties with dominant gain-of-function properties can explain the unusual overlap of symptoms in the homozygous carrier. Curiously, despite severely reduced availability of Na_V_1.4 channels in this patient, the gain-of-function properties of p.R1451L (i.e. enhanced recovery from inactivation) can still induce eyelid and EMG myotonia. Our data also suggests that hypoPP can be the predominant presentation of bi-allelic loss-of-function variants and that it can manifest without accompanying myasthenia.

Finally, coenzyme Q10, a component of the electron transport chain, ameliorated recurrent muscle weakness of the patients and increased the CMAP baseline amplitude value. The mechanism of action of coenzyme Q10 in improving the hypoPP symptoms is unclear but our data warrants consideration of its clinical benefits for hypoPP patients^36^.

## Materials and Methods

### Standard protocol approvals and patient consents

The study of family 1 was approved and all experiments were performed under the guidelines of the Institutional Ethics Committee of the Huashan Hospital. The patient and the family members provided written informed consent for clinical-genetic correlation studies.

For family 2, all procedures were performed as part of routine clinical care. The study was conducted under the ethics guidelines issued by our institution and approved by the London – Queen Square NRES Committee (reference07/Q0512/26), with written informed consent obtained from all participants for genetic and research studies.

### Electromyographic studies and exercise test protocols

For family 1, needle EMG studies were conducted in 11 muscles for each case. The LET and SET were performed following the Fournier protocol^37^. The ulnar nerve was stimulated supramaximally at the wrist, and the CMAP was recorded from the ADM every 30 s for 2 min to obtain a stable baseline amplitude value. The patient was required for forceful abduction of the small finger for 5 min with a 5-s rest period every 45 s. The CMAPs were recorded at 1-min intervals for the first 5 min and then at 5-min intervals for 40 min. The baseline-to-negative peak amplitude of each CMAP was expressed as a percentage of the baseline value before exercise. SET was performed on the small finger after the forceful abduction for 10 seconds, CMAPs were recorded 2 seconds immediately after the end of exercise and then every 10 seconds for 50 seconds.

### Screening of mutations by targeted next generation sequencing

For family 1 a targeted next generation sequencing panel comprising 10 ion channel genes and 245 primary myopathies/muscular dystrophies-related genes (Suppl. Table 1) was employed to screen the causal mutations. Genomic DNA from peripheral blood was extracted with High Pure PCR Template Preparation Kit (Roche, Basel,CH) according to the manufacturer’s instructions. DNA fragments were enriched by solution-based hybridization capture and followed by sequencing with an Illumina Miseq platform (Illumina, San Diego, CA, USA) with the 2 × 300 bp paired-end read module. The hybridization capture procedure was performed with the SureSelect Library Prep Kit (Agilent, Santa Clara, CA, USA). For further verification of the candidate mutations, we performed the Sanger sequencing with the DNA samples extracted from the patient and the parents. PCR was performed with GoldStar Taq DNA Polymerase (CWbiotech) according to standard protocol. PCR products were sequenced on an ABI3730xl DNA Analyzer (Applied Biosystems). Public databases including dbSNP138, 1000 Genome project, Exome Sequencing Project, ExAC, ClinicVar and HGMD were used to screen variants. Prediction of functional effect was evaluated by PolyPhen-2 and SIFT scores. To detect CNV (Copy number variant), sequencing depth of each region covered by the probes was calculated according to the alignment files. ExomeDepth Package was also used to find potential CNVs.

For family 2 genetic analysis of ion channel genes *SCN4A*, *CACNA1S*, *KCNJ2 and CLCN1* was performed at the Neurogenetics Unit, National Hospital for Neurology and Neurosurgery as provided by the Channelopathy Highly Specialized National Service for rare disease. Samples underwent Next-Generation Sequencing on an Illumina HiSeq following enrichment with an Illumina custom Nextera Rapid Capture panel (Illumina, Inc., San Diego, CA).

### Molecular Biology and cell preparation

Human and rat *SCN4A* constructs were used as templates for site directed mutagenesis using the QuickChange kit (Agilent Technologies). The target mutation p.R1451L (human)/p.R1444L (rat) and insert sequence integrity were verified by sequencing. *In vitro* transcription was performed with the mMessage mMachine kit (Ambion).

HEK293 cells were cotransfected with h*SCN4A* (500 ng) and GFP (50 ng) cDNAs using 1.5 µl Lipofectamine 2000 (Gibco) on a 1.9 cm^2^ dish.

*Xenopus laevis* ovarian lobes were obtained according to procedures approved by the UCL Biological Services and the UK Home Office. Oocytes were defolliculated with enzymatic digestion using Collagenase A (2 mg/ml, Roche) in oocyte Ringer (in mM: NaCl 82.5, KCl 2, MgCl_2_, HEPES 5, pH 7.5–6) and stored in modified Barth’s Solution (in mM: NaCl 87.1, KCl 1, MgSO_4_ 1.68, HEPES 10, NaNO_3_ 0.94, NaHCO_3_ 2.4, CaCl_2_ 0.88, pH 7.4) supplemented with penicillin (50 U/ml), streptomycin (50 μg/ml) and amikacin (100 μg/ml) at 14–18 °C. Selected oocytes were injected with RNA for rat *SCN4A* and rat *SCN1B* (~50 ng each).

### Electrophysiology

Gating pore currents through Na_V_1.4 were studied in *Xenopus* oocytes by two-electrode voltage clamp with a GeneClamp 500B amplifier, Digidata 1200 digitizer and pCLAMP™ software (Molecular Devices). Electrodes had a resistance of 0.1–0.7 MΩ when filled with KCl 3 M. Oocytes were bathed in a solution containing (in mM): 120 NaMeSO_4_, 1.8 CaSO_4_, 10 HEPES, pH 7.4. Currents were elicited using 300 ms voltage steps from −140 to +50 mV in 5 mV increments from a holding potential of −100 mV. Sampling frequency was 10 kHz. TTX 1–2 μM was added to the bath to block Na^+^ currents through the main channel pore and presence of gating pore currents analysed by replacing 50% of NaMeSO_4_ with guanidine sulfate. Gating pore currents were measured as the average steady state current in the last 50 ms of the test pulse and plotted against membrane voltage. Leak currents were measured by fitting a straight line to the current-voltage data in the range + 10 mV to +30 mV. The fitted line was extrapolated to cover the voltage range of the measurements and subtracted from the raw current data.

The properties of Na_V_1.4 main pore currents were studied with whole cell patch clamp recordings from HEK293 cells 48 hours post-transfection. Data were sampled at 50 Hz and filtered at 5 Hz using Axopatch 200B, Digidata 1440B and pClamp software (Molecular Devices). Electrodes (1–3 MΩ) were pulled from borosilicate glass and filled with the following intracellular solution (in mM): CsCl 145, NaCl 5, EGTA 10, HEPES 10 (pH 7.4). Bath solution was (in mM): NaCl 145, KCl 4, CaCl_2_ 1.8, MgCl_2_ 1, and HEPES 10 (pH 7.35). The calculated liquid junction potential of −4.4 mV was not corrected for. Series resistance was compensated ≥ 70% to keep the voltage error below 5 mV. Holding potential was −80 mV or −100 mV. Leak and capacitance subtraction was performed online using a -*P*/4 procedure for all protocols except for pulse trains and for slow inactivation. The voltage protocol to measure the voltage dependence of activation and fast inactivation consists of a 150 ms step to test voltages ranging from −150 mV to +50 mV in 10 mV increments, followed by a step to a tail voltage of −10 mV. Peak current in response to test voltages was used to analyse voltage dependence of activation. Peak current in response to the tail voltage was used to measure the voltage dependence of inactivation. Peak conductance of activation was derived by dividing the peak current by test voltage subtracted by reversal voltage. Reversal voltage was estimated for each individual cell by extrapolating a straight line fitted to the voltage range of +20 to +50 mV to I = 0 pA. Voltage of half-maximal activation or inactivation (V_1/2_) and the slope factor *K*, were measured by fitting with a Boltzmann equation (Y = A + (B-A)/(1 + exp((V-V_1/2_)/*K*)) where Y is conductance or current, and A and B are the minimum and maximum amplitudes of the fit. Time course of onset of open state fast inactivation was estimated using the same protocol by fitting a double exponential function to current decay. Only the main component of the double exponential function was analysed.

Time course of closed state inactivation was studied by applying pre-pulses of increasing duration to −60 mV or −80 mV, followed by a test pulse to 0 mV. Time course of recovery from fast inactivation was studied by inactivating the channels for 20 ms at 0 mV, applying recovery-pulses of increasing duration to −80 mV or −100 mV, before applying the second pulse to 0 mV. The peak current (closed state inactivation) or the ratio of peak currents in response to second and first test pulse (recovery from inactivation) is plotted against the duration of pre- or recovery-pulse.

For analysis of responses to repetitive stimulation, trains of 25 pulses of 2 ms duration to 0 mV were applied with varying frequencies, ranging from 20 Hz to 400 Hz.

Voltage dependence of slow inactivation was studied by applying 10 s pre-pulse steps to voltages ranging from −130 mV to +50 mV in 10 mV increments. This was followed by a 20 ms pulse to −100 mV to recover channels from fast inactivation and a test pulse to −10 mV. Peak currents in response to the test pulse were measured and plotted against pre-pulse voltage.

### Data analysis

Current records were analyzed using Clampfit 10.7, OriginPro 2016 and GraphPad softwares. All data is presented as mean ± SEM. Unpaired t tests were used for statistical comparison of biophysical properties (with Welch correction for parameters with unequal variance) or non-parametric (Mann-Whitney) tests if data was not normally distributed. One way ANOVA and Dunnet multiple comparisons test was used for gating pore current data. Significance was established at p < 0.05.

### Data availability

The datasets generated during and/or analysed during the current study are available from the corresponding author on reasonable request.

## Electronic supplementary material

Supplementery information
