# Supplementary material for: Hypokalaemic periodic paralysis and myotonia in a patient with homozygous mutation p.R1451L in NaV1.4
Source: Sci Rep. 2018 Jun 26;8:9714. doi: 10.1038/s41598-018-27822-2 (PMC6018793; doi:10.1038/s41598-018-27822-2)
Supplement: Supplementary file 1 — Supplementery information [file 41598_2018_27822_MOESM1_ESM.pdf]

Supplementary information to

Hypokalaemic periodic paralysis and myotonia in a patient with homozygous mutation p.R1451L in $Na_V1.4$

Sushan Luo[a,b,1], Marisol Sampedro Castañeda[c,1], Emma Matthews[c], Richa Sud[c], Michael G Hanna[c], Jian Sun[a], Jie Song[a], Jiahong Lu[a], Kai Qiao[d,] *, Chongbo Zhao[a,e,]*, Roope Männikkö[c,]**

[a]*Department of Neurology, Huashan Hospital, Fudan University, Shanghai, China 200040*

[b]*Department of Neurology, North Huashan Hospital, Fudan University, Shanghai, 200003, China*

[c]*MRC Centre for Neuromuscular Diseases, Department of Molecular Neuroscience, UCL Institute of Neurology, London, WC1N 3BG, UK*

[d]*Department of Clinical Electrophysiology, Institute of Neurology, Huashan Hospital, Fudan University, Shanghai, China 200040*

[e]*Department of Neurology, Jing'an District Center Hospital of Shanghai, Shanghai, China 200040*

Supplementary table S1. Genes included in the panel for targeted next generation sequencing.

**Skeletal ion Channel related genes**						
ALG13	CACNA1S	CAV3	CLCN1	KCNA2	KCNE3	KCNJ18
KCNJ2	SCN4A	TMEM43				
**Primary myopathies related genes**						
ABHD5	ACADL	ACADM	ACADS	ACADVL	ACTA1	ACTN3
ACVR1	ADCK3	AGK	AGL	AGRN	ALDOA	ALG14
ALG2	ANO5	ARFGAP2	ATP2A1	B3GALNT2	B3GNT1	B4GALT1
B4GAT1	BAG3	BIN1	BOLA3	C10orf2	CAPN3	CASQ1
CCDC78	CELF1	CFL2	CHAT	CHCHD10	CHKB	CHRNA1
CHRNB1	CHRND	CHRNE	CHRNG	CKM	CMYA5	CNBP
CNTN1	COL12A1	COL6A1	COL6A2	COL6A3	COLQ	COQ2
COQ6	COQ9	CPT2	CRYAB	CSRP3	DAG1	DES
DMD	DMPK	DNAJB6	DNM2	DOK7	DOLK	DPAGT1
DPM1	DPM2	DPM3	DUX4	DYSF	EMD	ENO3
EPM2A	ETFA	ETFB	ETFDH	ETHE1	FBXO32	FHL1
FHL2	FKRP	FKTN	FLNC	GAA	GBE1	GFPT1
GMPPB	GNE	GYG1	GYS1	HACD1	HADH	HINT1
HNRNPDL	HSPG2	ISCU	ISPD	ITGA7	ITGA9	KBTBD13
KLHL40	KLHL41	KLHL9	KY	LAMA2	LAMB2	LAMP1
LAMP2	LARGE	LDB3	LDHA	LDHB	LIFR	LIMS2
LMNA	LPIN1	LRP4	MATR3	MBNL1	MBNL2	MBNL3
MEGF10	MICU1	MSTN	MTM1	MTMR14	MUSK	MYBPC1
MYBPC2	MYBPC3	MYH1	MYH2	MYH3	MYH4	MYH7
MYH8	MYL1	MYL10	MYL12A	MYL12B	MYL2	MYL3
MYL4	MYL5	MYL6	MYL6B	MYL7	MYL9	MYLIP

MYLK	MYLK2	MYLK3	MYLK4	MYLPF	MYOM1	MYOM2
MYOM3	MYOT	MYOZ1	MYOZ2	MYOZ3	MYPN	NBR1
NEB	NEBL	NHLRC1	NTRK1	OBSCN	OBSL1	OPA1
ORAI1	PABPN1	PDLIM3	PDLIM5	PDLIM7	PDSS1	PDSS2
PFKM	PGAM2	PGK1	PGM1	PHKA1	PLEC	PLEKHG4
PLN	PNPLA2	POLG	POMGNT1	POMGNT2	POMK	POMT1
POMT2	PRKAG2	PTRF	PUS1	PYGM	QDPR	RAPSN
RBCK1	RRM2B	RYR1	SEPN1	SGCA	SGCB	SGCD
SGCG	SLC22A5	SLC25A20	SLC25A4	SMCHD1	SOX10	SPEG
SQSTM1	SRF	STIM1	SUCLA2	SYNE1	SYNE2	SYNE3
SYNPO2	SYT2	TCAP	TIA1	TK2	TMEM5	TMOD3
TNNC1	TNNC2	TNNI1	TNNI2	TNNI3	TNNT1	TNNT3
TNPO3	TOR1AIP1	TPM1	TPM2	TPM3	TRAPPC11	TRIM32
TRIM55	TRIM63	TTN	TTR	VCP	VMA21	YARS2

Supplementary Figure S1. Rate of closed state inactivation depends on holding voltage.

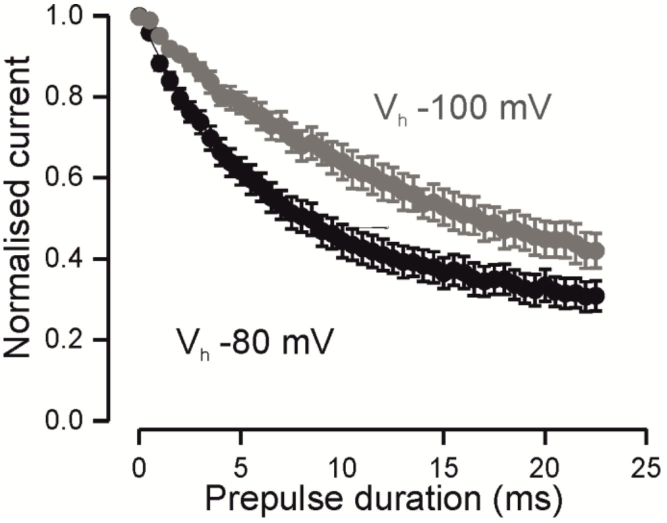

A. Rate of closed state inactivation at -60 mV from V_h -100 mV (grey) or from V_h -80 mV (black) for wild-type channels. Current in response to test voltage is plotted against the time at pre-pulse voltage. Data are normalised to maximum current in response to test pulse. Voltage protocol is shown in Fig 5G.
